# 14. VIOLENCE IN SCHIZOPHRENIA: PREVALENCE, MEASUREMENT, PREDICTION AND PREVENTION

**DOI:** 10.1093/schbul/sby014.053

**Published:** 2018-04-01

**Authors:** Mark Weiser

**Affiliations:** Sheba Medical Center

## Abstract

**Overall Abstract:** Most patients with schizophrenia and bipolar disorders (severe mental illness, SMI) are not violent in their lifetimes, however, a minority of patients are violent at some points in the course of their illness. As the illness appears relatively early in life, and typically runs a chronic course, the number of violent incidents caused by patients can be considerable in some cases. Due to the stigma toward SMI, the media often emphasize reporting of these incidents, which fuel the stigma even more. Although violent behavior is a common cause of concern for patients, their families and clinicians, it is not often discussed in scientific meetings. The purpose of this symposium is to bring this relatively neglected, but very important topic into the spotlight in SIRS, in order to summarize the latest evidence for clinicians and researchers, and to foster new work on reducing these risks of violence.

Dr. Weiser will present an overview of the prevalence of violent behavior in patients with SMI, and will present a population-based, case-control study from Israel, showing increased rates of violent crime in patients with SMI, particularly in female patients and patients who abuse drugs. Secondary analyses will show increased rates of violent behaviour in siblings of patients as well.

Dr. Fazel will present a systematic review on the prognostic (or predictive) accuracy of structured ways to assess violence risk in patients with severe mental illness, and present new work on a scalable and potentially useful predictive model of violent behaviour based on 75,000 patients in Sweden.

Dr. Nijman will present a model based on patient, ward and staff variables focused on the causes and triggers of aggressive behavior on (locked) psychiatric wards. Based on this model, a number of preventive measures can be formulated. At the patient level, the administration of anti-psychotic medication is used to reduce the negative cognitive schemes and delusional thoughts that are depicted in the center of the model. A more novel intervention at the patient level may be the additional administration of nutritional supplements with (among others) high levels of omega 3 fatty acids. The results of two Dutch studies on this topic will be briefly presented in the lecture, among which a RCT on the effects of the use of nutritional supplements on aggressiveness. On the staff level, the use of short-term (daily) risks assessments by the ward nursing staff, among others by means of the six item BrØset Violence Checklist (BVC), has been found to reduce aggressiveness as well as the use of coercive measures on psychiatric wards in two cluster randomized RCTs. On the ward level, studies indicate that aggression on psychiatric wards can be reduced by preventing overcrowding on psychiatric wards, and by providing more space and privacy to the patients.

Dr. Torrey will present data on rates of re-arrest in patients with SMI, showing that the average five-year re-arrest rate is approximately 40% for those released from psychiatric hospitals and 60% for those released from jails or prisons, and will present comparison data from other countries. He will then present data on the effect of extended conditional release, Forensic Assertive Community Treatment (FACT) teams, and Psychiatric Security Review Boards on re-arrest rates.

